# Tumor endothelial ELTD1 as a predictive marker for treatment of renal cancer patients with sunitinib

**DOI:** 10.1186/s12885-020-06770-z

**Published:** 2020-04-22

**Authors:** Marjut Niinivirta, Maria Georganaki, Gunilla Enblad, Cecilia Lindskog, Anna Dimberg, Gustav J. Ullenhag

**Affiliations:** 1 Department of Immunology, Genetics and Pathology, Uppsala University, 75185 Uppsala, Sweden; 2 Department of Oncology, Entrance 78, Uppsala University Hospital, 751 85 Uppsala, Sweden; 3 Department of Immunology, Genetics and Pathology and Science for Life Laboratory, Uppsala University, 751 85 Uppsala, Sweden

**Keywords:** ELTD1, Predictive marker, Renal cancer, Tissue microarray, Tyrosine kinase inhibitor

## Abstract

**Background:**

Patients with metastatic renal cell cancer (mRCC) are commonly treated with the tyrosine kinase inhibitor sunitinib, which blocks signalling from vascular endothelial growth factor (VEGF) - and platelet-derived growth factor-receptors, inhibiting development of new blood vessels. There are currently no predictive markers available to select patients who will gain from this treatment. Epidermal growth factor, latrophilin and seven transmembrane domain-containing protein 1 (ELTD1) is up-regulated in tumor endothelial cells in many types of cancer and may be a putative predictive biomarker due to its association with ongoing angiogenesis.

**Methods:**

ELTD1, CD34 and VEGF receptor 2 (VEGFR2) expressions were analysed in tumor vessels of renal cancer tissues from 139 patients with mRCC using immunohistochemistry. Ninety-nine patients were treated with sunitinib as the first or second-line therapy. Early toxicity, leading to the termination of the treatment, eliminated 22 patients from the analyses. The remaining (*n* = 77) patients were included in the current study. In an additional analysis, 53 sorafenib treated patients were evaluated.

**Results:**

Patients with high ELTD1 expression in the tumor vasculature experienced a significantly better progression free survival (PFS) with sunitinib treatment as compared to patients with low ELTD1 expression (8 versus 5.5 months, respectively). The expression level of CD34 and VEGFR2 showed no correlation to sunitinib response. In sorafenib treated patients, no association with ELTD1 expression and PFS/OS was found.

**Conclusions:**

Our results identify tumor vessel ELTD1 expression as a positive predictive marker for sunitinib-treatment in patients suffering from mRCC. The negative results in the sorafenib treated group supports ELTD1 being a pure predictive and not a prognostic marker for sunitinib therapy.

## Background

Treatment with tyrosine kinase inhibitors (TKIs) improves survival for patients with metastatic renal cell cancer (mRCC) [1]. One of the first and most commonly used TKI, sunitinib, increases progression free survival (PFS) with a median of 6 months compared to interferon alpha (IFN-α) [2]. However, not all patients benefit from treatment with TKIs. Side effects, ranging from mild reversible to chronic toxicity, could be avoided and the costs of the medication could be reduced if predictive biomarkers for TKI treatment were available.

There are several established prognostic factors for mRCC patients and these were updated by Heng in 2009. Hengs criteria include Karnofsky performance status, hemoglobin, calcium, time from diagnosis to treatment, neutrophils and platelet counts [3]. Predictive factors indicate the sensitivity or resistance to a specific medication. There are currently no established predictive markers for TKI treatment.

Most studies trying to find predictors have analysed the connection between different circulating proteins in serum and response to sunitinib therapy [4–7]. Tissue microarray (TMA) analysis enables direct investigation of protein expression in malignant cells and stroma, but only a few studies have been reported. For hypoxia-inducible factor 1α (HIF-1α), CD31, vascular endothelial growth factor (VEGF) receptors, CA9, Ki67 and platelet-derived growth factor receptor α (pPDGFRα) associations with response to sunitinib therapy have been demonstrated [8, 9]. In another and larger TMA-based study, tumoral expression of programmed death ligand 1 (PD-L1) or PD-L1 plus tumor infiltrating CD8+ T-cells were correlated to significantly shorter PFS and overall survival (OS) in patients treated with sunitinib or pazopanib [10]. We have previously reported TMA-studies indicating that cubilin (CUBN) and annexin A1 (ANXA1) expressed in the tumor cells are predictive markers in mRCC patients treated with sunitinib and sorafenib [11, 12].

In about 60–75% of clear cell renal cell cancers (RCC) the tumor suppressor gene von Hippel-Lindau (VHL) is inactivated leading to accumulation of the hypoxia-inducible factor (HIF) which leads to overexpression of VEGF and PDGF [13]. Vascular endothelial growth factor and PDGF are growth factors stimulating angiogenesis, tumor spread and tumor growth [14, 15]. Tumor-associated vessels are larger than normal vessels and leaky which leads to high interstitial fluid pressure (IFP) and swelling in and around tumor tissues. The nutrient and oxygen delivery is poor leading to hypoxia within the tumor which stimulates production of pro-angiogenetic factors and continued development of abnormal vasculature [16]. Tyrosine kinase inhibitors, such as sunitinib, sorafenib and pazopanib, block VEGF- and PDGF-receptors and thereby inhibit development of new pathological blood vessels [17].

Epidermal growth factor (EGF), latrophilin and seven transmembrane domain-containing protein 1 (ELTD1), also named as adhesion G protein-coupled receptor L4 (ADGRL4), is thought to play a role in pathological angiogenesis. It belongs to the adhesion G-protein-coupled receptor (GPCR) superfamily [18]. ELTD1 expression is part of a recently identified gene signature commonly associated with angiogenesis in human tumors, and is frequently upregulated in tumor-associated endothelial cells (EC) in human cancer, including, renal carcinoma [19, 20]. Higher intratumoral EC ELTD1 expression was significantly correlated with smaller tumor size and better prognosis in mRCC patients [19]. These findings indicate that ELTD1 expression is regulated during tumor angiogenesis in RCC, and is therefore a putative biomarker for response to anti-angiogenic therapy.

The aim of this study was to explore the potential value of assessing the expression of ELTD1, CD34 and VEGFR2 in tumor vessels to predict benefit of sunitinib treatment in mRCC patients.

## Methods

### Patients

**Fig. 1:**
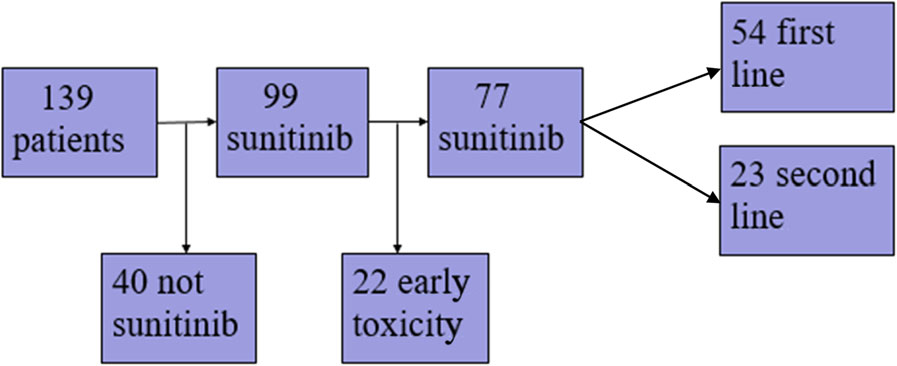
A flow chart presenting enrollment of the sunitinib treated patients in this study

The local Research Ethics Committee in Uppsala granted approval for the study (2009/139) and patients still alive gave their written informed consent. The TMA cohort and treatment characteristics have been described previously [11, 12, 21] (Table 1) [12]. In short, the TMA consists of 139 cases of RCC diagnosed 2006–2010. All the patients underwent nephrectomy at diagnosis and later on were treated with anti-cancer therapy for mRCC. A total of 99 patients were treated with sunitinib in the first or second line setting, while 53 received treatment with sorafenib. A flow chart presenting enrollment of the sunitinib treated patients is demonstrated in Fig. 1.

**Table 1:** Treatment characteristics

Sunitinib treatment	Total ***n*** = 99
Sunitinib first line, n (%)	70 (71)
Sunitinib second line, n (%)	29 (29)
Side effects leading to discontinuation of treatment, n (%)	22 (22)
First line	16
Second line	6
Treated until progression/end of follow-up, n (%)	77 (78)
First line	54
Second line	23
Median PFS, months (range)	7 (0,5–34)
First line	7,8 (0,5–34)
Second line	6 (1–24)
Still under treatment, n (%)	11 (14)

Treatment characteristics for renal cancer patients treated for metastatic disease with sunitinib in the first- and second-line setting [12].

Patient and tumor characteristics in our cohort have been described previously (Table 2) [12].

**Table 2:** Patient and tumor characteristics

Patient cohort	Total ***n*** = 77
Gender, n (%)	
Male	53 (69)
Female	24 (31)
Age at diagnosis, years	
Median (range)	62 (40–76)
Age at metastatic disease, years	
Median (range)	64,5 (40–77)
Histologictype, n (%)	
Clear cell	68 (88)
Papillary	2 (3)
Mixed phenotype	2 (3)
Unknown	5 (6)
Local disease at diagnosis, n (%)	36 (47)
Metastatic disease at diagnosis, n (%)	41 (53)
Time to metastasis, years	
Median (range)	2 (0–18)
Metastasis during first year, n (%)	14 (39)
Metastasis after first year, n (%)	22 (61)
Alive, n (%)	16 (21)
Dead, n (%)	61 (79)

Clinical characteristics of renal cancer patients treated for metastatic disease with sunitinib in the first- and second-line setting [12].

### TMA generation, immunohistochemical methods and slide scanning

TMA, immunohistochemistry and slide scanning were essentially performed in accordance to standards used in the Human Protein Atlas (www.proteinatlas.org) and the procedure has been described previously [11, 12, 21–23]. The TMA in our study consists of core biopsies from the primary renal tumor. The antibody concentrations were tested according to experience from testing and validating antibodies within the Human Protein Atlas Projects using a test TMA containing normal and tumor tissues. The proteins of interest are known to display a vascular expression pattern and to be enriched in many tumor vessels, providing internal positive and negative controls within the TMA.

The staining and immunohistochemical procedure has been previously in detail described [11, 12, 21]. In this study we used the primary rabbit polyclonal antibody towards ELTD1 (HPA025229, Atlas Antibodies, Stockholm, Sweden), CD34 (CAB000018, Dako Cat#7165, Agilent (Formerly DakoCytomation) and VEGFR2 (CAB004028, Cell Signaling Technology Cat#2479, Cell Singaling Technology, Inc).

### Image analysis

High-resolution images from each individual TMA core underwent colour deconvolution based on the H-DAB setting of the Fiji software to separate the DAB from the Hematoxylin staining [24]. The ELTD1-, CD34- and VEGFR2-positive area percentage of each TMA core was measured from the generated DAB images by a CellProfiler pipeline based on color-thresholding [25].

### Statistical methods

Statistical analyses (Kaplan-Meier method, log-rank test) were performed using STATISTICA program (version 2012). A two sided *p*-value < 0.05 was defined as statistically significant.

## Results

### High vascular expression of ELTD1 in mRCC predicts a favourable response to sunitinib treatment

**Fig. 2:**
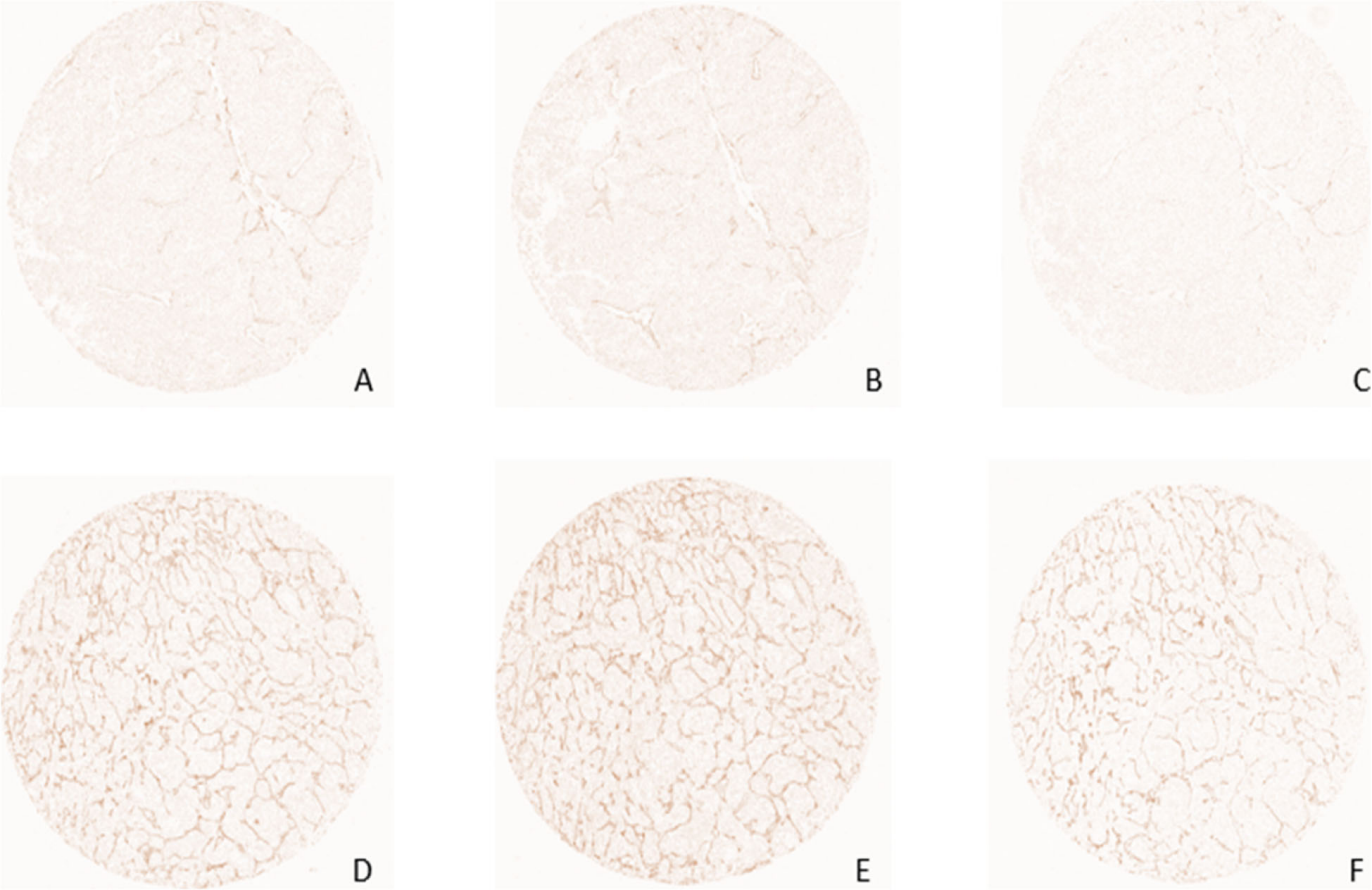
Representative immunohistochemical images of ELTD1, CD34 and VEGFR2. Images demonstrating low ELTD1 (**a**), CD34 (**b**), VEGFR2 (**c**) and high ELTD1 (**d**), CD34 (**e**) and VEGFR2 (**f**) staining in the primary renal tumor vasculature in patients later treated for metastatic disease with sunitinib in the first or second line setting

Tissue microarrays representing tumor cores from 99 patients diagnosed with mRCC that had undergone nephrectomy and were treated with sunitinib in first or second line therapy were stained using ELTD1-specific antibodies as described in materials and methods. ELTD1 expression was exclusively noted in tumor vessels, while other stromal cells and the tumor cells were uniformly negative (Fig. 2). A varying proportion of vessels within each core expressed ELTD1, while the intensity of staining was fairly similar in positive vessels. The area of ELTD1-expressing tumor vessels in each core was determined through digital analysis of scanned images as described above, and the percentage of ELTD1 positive area as compared to the total tumor area was calculated. To determine if vascular expression of ELTD1 could predict response to sunitinib therapy, patients were classified into two groups according to the median expression of ELTD1. The median expression was 1.1 (%) and the expression range 0.3–5.6 (%). Patients with < 1.1 (%) expression were categorized as ELTD1 low and patients with ≥1.1 (%) as ELTD1 high. The primary end-point of the study was PFS, calculated as the time from treatment initiation to the time of clinical and/or radiological progression, treatment termination due to toxicity, death or end of follow up and the second end-point was OS, calculated from the diagnosis of mRCC, in regard with ELTD1 expression. Using the cut-off value for staining described above, 39/77 (50%) cases were ELTD1 high.

**Fig. 3:**
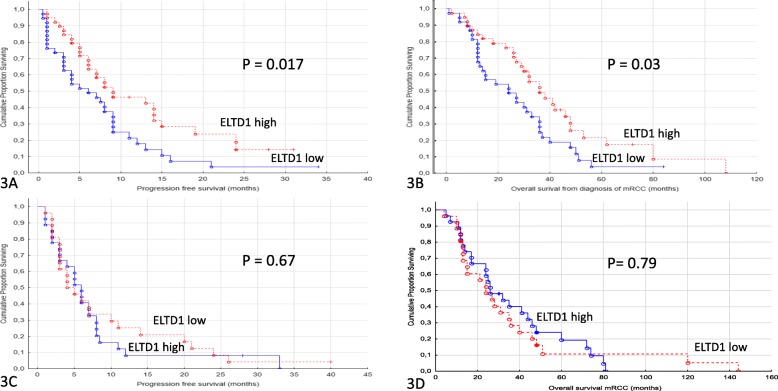
**a** and **b**: Progression free survival (3A) and overall survival (3B) and expression of ELTD1 in sunitinib treated patients. Patients treated for metastatic renal cell cancer with sunitinib in the first or second line setting (*n* = 77) comparing ELTD1 low versus ELTD1 high. **c** and **d**: Progression free survival (3C) and overall survival (3D) in sorafenib treated and expression of ELTD1. Patients treated for metastatic renal cell cancer with sorafenib in the first or second line setting (*n* = 53) comparing ELTD1 low versus ELTD1 high

Patients with a higher area percentage of ELTD1-expressing tumor vessels had a significantly better PFS (*p* = 0.017, Fig. 3a). We observed that the median sunitinib treatment period for patients with a high ELTD1-positive vessel area was 8 months (range 1–31 months) as compared to 5,5 months for patients with a low ELTD1-positive vessel area (range 0.5–34). Patients with a high ELTD1-positive vessel area had a significantly better OS (*p* = 0.03, Fig. 3b). The ELTD1 high group had a median OS of 31 months (range 2–108 months) while the ELTD1 low group had a median OS of 24 months (range 1–84 months). To determine if tumor vessel expression of ELTD1 generally predicted response to TKI-therapy, we analysed TMA cores from patients treated with sorafenib in the first or second line setting (*n* = 53). ELTD1-positive area fraction was not associated with either PFS or OS (*p* = 0.67/0.79) in these patients (Fig. 3c and d).

### Vessel area does not predict response to sunitinib treatment in mRCC

**Fig. 4:**
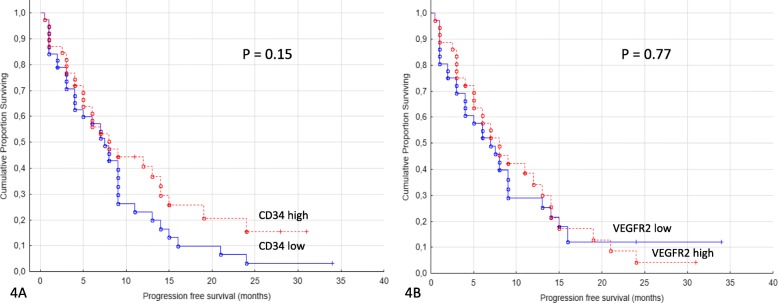
**a** and **b**: Progression free survival in sunitinib treated and expression of CD34 and VEGFR2. Patients treated for metastatic renal cell cancer with sunitinib in the first or second line setting (*n* = 77) comparing CD34 low versus CD34 high and VEGFR2 low versus VEGFR2 high

To determine if the area fraction of ELTD1-positive vessels simply reflected vessel density, the expression of pan-endothelial markers CD34 and VEGFR2 were assessed. CD 34 is widely expressed in both normal and tumor vessels and VEGFR2 is the most prominent receptor for VEGF-induced blood vasculature development and also one of the targets for sunitinib [26]. CD34 and VEGFR2 expression were uniformly expressed in tumor vessels and not present in other cell types within the tumor microenvironment (Fig. 2). The area fraction of CD34 and VEGFR2 within each core was calculated. Patients were categorized into CD34 and VEGFR2 high versus CD34 and VEGFR2 low groups according to the median expression (0.7 and 0.9%, respectively). There were no significant correlation between either CD34-positive area fraction or VEGFR2-positive area fraction and PFS/OS (*p* = 0.15/0.77 for PFS and 0.62/0.85 for OS) for sunitinib treated patients (Fig. 4a and b).

## Discussion

About 20% of RCC patients suffer from a metastatic disease already at the time of diagnosis [27]. Prognosis has been very poor for these patients throughout the years. No chemotherapy nor radiotherapy has been successful [28] and only a minority of the patients respond to cytokine treatment with IFN-α and IL-2 used earlier [29]. Novel molecular agents such as TKIs have changed the standard of treatment for mRCC and prolonged the survival [1]. Recently, the combination of the anti PD-1 antibody nivolumab and cytotoxic T-lymphocyte associated protein 4 (CTLA4) ipilimumab was registered as a first line treatment for intermediate and poor-risk mRCC patients based on a positive study comparing this combination with single sunitinib [30]. However, all patients do not benefit from this specific immunotherapy and many patients develop adverse side effects leading to discontinued therapy. Therefore, TKIs are still considered as one of the cornerstones in the treatment arsenal for mRCC patients. Predictive markers are needed to use TKIs more accurately and select individuals who will benefit from the treatment. No such markers are in clinical use yet.

Some serum proteins have been suggested as predictors for treatment with TKIs in mRCC patients, especially for sunitinib treatment. In a few (*n* = 21) sunitinib medicated patients, increased baseline levels of tumor necrosis factor α (TNF-α) and metalloproteinase-9 (MMP-9) were associated with non-responders and reduced time to progression (TTP) and OS [6]. Another sunitinib study (*n* = 85) demonstrated that elevated baseline levels of serum VEGF and neutrophilgelatinase-associated lipocalin (NGAL) correlated to higher relative risk of progression [7]. Some clinical side-effects related to TKIs, such as development of hypertension and hand-foot skin reaction correlate with better response and improved OS [31–33]. Hence, instead of predicting the benefit they have a role as early evaluators of the treatment.

Putative predictive tumoral proteins can be analysed with TMAs, where all cores are stained at the same time and under same conditions. In one study, patients with elevated tumor expression of HIF-α or expression of VEGFR3 in tumor vessels experienced improved PFS while treated with sunitinib. In the same study a low CA9 score was correlated to a shorter OS [8]. Tumoral expression of PD-L1 or PD-L1 plus tumor-infiltrating CD8+ T cell counts predicted shorter PFS and OS for patients treated with sunitinib or pazopanib in a TMA of mRCC cases [10].

The function of ELTD1 in tumor vessels has not been fully elucidated, but a role as a regulator of vascular sprouting has been proposed [19]. In renal cancers (*n* = 157), higher ELTD1 expression on tumor-associated ECs was significantly correlated with improved survival. Surprisingly, silencing ELTD1 inhibited tumor growth in experimental models. Upon in vivo ELTD1 knockdown, microvascular density (MVD) was significantly reduced, hypoxia and apoptosis of ECs increased and cell proliferation was inhibited [19].

The advantage of using imaging analyses for scoring is that it is more objective and quantitative than manual assessment [34]. We found that patients with high ELTD1 staining in their primary tumor vasculature had a greater benefit from sunitinib treatment in terms of a prolonged PFS. ELTD1, expressed on ECs, may act as an indicator of activated angiogenesis. As the goal of the sunitinib-treatment is to block new pathological vessels to grow, high ELTD1 expression in tumor vasculature could indicate patients with better likelihood to respond to this treatment, as our results demonstrate. Hence, there is a scientific basis for our finding.

In a subgroup analysis we separated patients having received sunitinib up front or as a second line treatment. The difference in PFS remained significant in the group having received sunitinib in the first line (*n* = 70) while the same correlation could not be recalled for the fewer mRCC patients (*n* = 29) who were treated with sunitinib after progression (data not shown). The sensitivity of the tumor to sunitinib therapy might be changed by the primary treatment and our subgroup analysis may indicate ELTD1 being a predictor limited only to the first line therapy. Another possible explanation for these findings is that a true difference also for the second line treated group may not be relived because of the limited number of patients.

In addition to PFS we observed also significantly longer OS in sunitinib treated patients with high ELTD1 vessel staining compared to the patients with low expression of ELTD1. The gain seen in PFS could lead to a prolonged OS. However, OS may also have been affected by other therapeutic regimens. Another established end point in cancer studies is objective response (OR). However, the benefit from TKIs is often not captured by OR but better by evaluating PFS as in the present study.

The level of expression of CD34 in the tumors did not correlate with benefit of sunitinib treatment. Since CD34 is a marker which is homogenously expressed on vascular endothelial cells, we conclude that the number of vessels is not predictive for sunitinib treatment. Furthermore, neither VEGFR2 which is a target for sunitinib was associated with response to the treatment.

We found no correlation between expression of ELTD1 and PFS or OS in sorafenib treated patients. The survival curves in this subanalysis do not show even a trend to separate and furthermore, no significant survival differences were demonstrated for the whole group**.** This confirms ELTD1 being a pure predictive and not a prognostic marker.

Combining ELTD1 with another biomarker might enhance the predictive value. We have previously shown the value of combining different biomarkers [11, 12].

There are a couple of limitations in the present study. First, potential heterogeneity between the primary tumors and metastases could not be assessed since there were no available metastatic tissues. Second, though the current study is one of the largest predictive marker studies reported for RCC patients, a higher number would have been desirable to yield more reliable results. The retrospective design does not affect our results as the aim of the study was to investigate a putative predictor, not yet another prognostic marker.

## Conclusions

To our knowledge, the present study on the angiogenetic marker ELTD1 is the first to examine its predictive value in mRCC patients treated with TKIs. In summary, in this real world study we show that high expression of ELTD1 on the tumor vessels is a potential predictive marker for sunitinib treatment. High ELTD1 staining on primary tumor vessels was in our material associated with significantly longer PFS and OS which is in line with ELTD1s role. A prognostic value of ELTD1 was ruled out since no association between expression of ELTD1 and PFS/OS was found in the sorafenib-treated group. Further studies are needed to confirm our results before ELTD1 can be routinely used to select patients for sunitinib treatment.

## Data Availability

The datasets used and/or analyzed during the current study are available from the corresponding author on reasonable request.

## Abbreviations

ANXA1: Annexin A1
CUBN: Cubilin
EC: Endothelial cell
ELTD1: Epidermal growth factor, latrophilin and seven transmembrane domain-containing protein 1
HIF: Hypoxia-inducible factor
IF- α: Interferon alfa
MMP-9: Metalloproteinase-9
mRCC: Metastatic RCC
mTOR: Mammalian target of rapamycin
NGAL: Neutropfilgelatinase-associated lipocalin
OS: Overall survival
PDGF: Platelet-derived growth factor
PD-L1: Programmed death-1 ligand
PFS: Progression free survival
RCC: Renal cell carcinoma
SciLifeLab: Swedish Science for Life Laboratory
TKI: Tyrosine kinase inhibitor
TMA: Tissue microarray
TNFα: Tumor necrosis factor α
VEGF: Vascular endothelial growth factor
VHL: Von Hippel-Lindau
